# 

**Published:** 1897-07

**Authors:** 


### CONTENTS.

ORIGINAL COMMUNICATIONS—

CLINICAL NOTES ON OBSTETRIC SURGERY. By Joseph Eve Allen, M.D., Augusta, Ga. 289
CAUSE AND PREVENTION OF CONSUMPTION—IS IT A CURABLE DISEASE? By J. S. Todd, M.D., Atlanta, Ga. .......... 296
INFANT MORTALITY, ITS PRIME CAUSE AND PREVENTION. By E. Van Goidtsnoven, A.M., M.D., Atlanta, Ga .......... 303
REMARKS ON FISTULA IN ANO. By Wm. S. Goldsmith, M.D., Atlanta, Ga .......... 314
LARYNGEAL DIPHTHERIA TREATED BY ANTITOXIN AND CALOMEL FUMIGATION. By E. C. Levy, M.D., Richmond, Va .......... 319

SOCIETY REPORTS—

ATLANTA SOCIETY OF MEDICINE .......... 323

CORRESPONDENCE—

NOTES FROM LONDON .......... 327
A BILL TO REGULATE THE EMPLOYMENT OF, AND PAY FOR, MEDICAL EXPERT TESTIMONY .......... 329
ACUTE TONSILLITIS .......... 331

EDITORIAL—

JUBILEE MEETING OF AMERICAN MEDICAL ASSOCIATION .......... 332
VIVISECTION IN THE DISTRICT OF COLUMBIA .......... 335

SELECTIONS AND ABSTRACTS .......... 336
MEDICAL ITEMS .......... 350
BOOK REVIEWS .......... 357
MEDICAL ITEMS—CONTINUED .......... x, xii, xiv, xvi, xvii, xviii, xix
ADVERTISERS' INDEX TO ADVERTISEMENTS .......... xx
CLASSIFIED INDEX TO ADVERTISEMENTS .......... xxxix

